# Common Features in Patients With Superior Canal Dehiscence Declining Surgical Treatment

**DOI:** 10.14740/jocmr2105w

**Published:** 2015-03-01

**Authors:** Lina Zahra Benamira, Anastasios Maniakas, Musaed Alzahrani, Issam Saliba

**Affiliations:** a Montreal University Hospital Center (CHUM), University of Montreal, Montreal, Quebec, Canada

**Keywords:** Superior canal, Dehiscence syndrome, Hyperacousis, Autophony, Occulophony

## Abstract

**Background:**

Superior canal dehiscence (SCD) is a benign condition in which a surgical treatment may be considered depending on the patients’ tolerance of their symptoms. In this study, we aim to identify driving factors behind the patients’ choice of a surgical management over watchful waiting.

**Methods:**

Sixty-two patients with cochlear and/or vestibular symptoms and a temporal bone high-resolution CT (HRCT) scan showing SCD were included in the study. All patients have been offered either surgical management or watchful waiting.

**Results:**

Of these, 28 elected surgery and 34 declined it. The operated group showed more cochlear (6.6 vs. 2.4) symptoms than the non-operated group (P < 0.001) except for hypoacousis, but no significant difference (P = 0.059) was found for the number of vestibular symptoms between both groups (3.4 vs. 1.1). Footstep and eating hyperacousis were both present in 57.1% of operated vs. 3% of non-operated patients (P < 0.001). Oscillopsia with effort and with walking was found in 50% and 35.7% of operated patients, respectively, but none in the non-operated group (P < 0.001). Hearing tuning fork at malleolus and Valsalva and pneumatic speculum induced vertigo showed a statistically significant difference between the two groups (P = 0.003, P < 0.001, P = 0.010 respectively). Cervical vestibular-evoked myogenic potential (cVEMP) thresholds, air and bone conduction thresholds, and mean air-bone gap (ABG) were similar in the two populations (P > 0.05). The average dehiscence size was 4.7 mm (2.0 - 8.0 mm) and 3.8 mm (1.3 - 7.7 mm) in the operated and non-operated patients, respectively (P = 0.421).

**Conclusions:**

The natures of cochleovestibular signs and symptoms were shown to be key factors in patients’ choice of a surgical management whereas paraclinical tests seem to be less significant in the patients’ decision for a surgical treatment.

## Introduction

In 1998, Minor first reported a syndrome in which a dehiscence of the bone overlying the superior semicircular canal (SSC) induces effects on both vestibular and auditory function. Creation of a “third mobile window” disturbing the endolymphatic movement in the bony labyrinth appears to be the main pathophysiologic mechanism of superior canal dehiscence syndrome (SCDS). The most common vestibular manifestations are sound- and/or pressure-induced vertigo and oscillopsia. As for auditory function, patients often show signs of conductive hyperacousis, defined as an increased sensitivity to bone conducted sounds. The latter manifests as autophony or unusual awareness of sounds such as one’s heel strike, heartbeat or even eye movements. Hearing loss generally completes the clinical presentation [1]. Diagnosis of SCDS relies on clinical findings, vestibular-evoked myogenic potential (VEMP) thresholds and radiologic findings of a defect in the bony roof overlying the SSC. Cervical VEMPs (cVEMP) are short-latency inhibitory muscular responses to intense sound and vibration stimulation. They are recorded over the ipsilateral sternocleidomastoid muscle while the latter is contracted [2]. The standard radiologic evaluation of patients suspected of having superior canal dehiscence (SCD) is a fine-cut (0.5- to 0.6-mm collimation) temporal bone high-resolution CT (HRCT) with the reconstruction of images parallel to the plane (Poschl’s view) of the SSC [3, 4]. The impact of SCD on the above diagnostic tests has been widely studied. We know for instance that thresholds for eliciting cVEMP using air-conducted sounds are usually lowered in the symptomatic ear when compared with normal controls and appear to normalize on canal plugging [5-8]. Moreover, patients with a dehiscence equal to or larger than 3.0 mm consistently show an air-bone gap (ABG) on audiometric tests [3, 6, 9-11].

Even though the optimal approach has yet to be determined, the surgical management of SCD is mainly focused upon closing the supernumerary “third mobile window” in the SSC. The traditional middle cranial fossa approach has been shown by our group and others to result in a significant improvement of patients’ preoperative symptoms [12]. Nevertheless, performing a craniotomy is not without risk, as complications such as facial paralysis, cerebrospinal fluid leak and intracranial bleeding can occur [13]. Thus, many otolaryngologists question the idea of subjecting patients to the risks of a surgery, especially when the complaints are limited to a few tolerable symptoms. That being said, once all the risks and benefits are explained, the decision lies in the hands of the patients who then weigh the risks and benefits with regard to the impact on their quality of life (QOL). To our knowledge, the features of the subpopulation of SCD patients who do not undergo a surgical treatment have never been explored. In this study, we wish to further expand our understanding of this syndrome by 1) presenting the features of this subpopulation of SCD patients and comparing it to a population of operated patients and 2) possibly highlighting a cluster of SCDS severity factors driving the need for surgery.

## Patients and Methods

We conducted a retrospective study of patients who underwent a temporal bone HRCT on the base of clinically suspected SCDS at our tertiary care center between February 2007 and July 2012. Approval by the local research ethics committee was obtained prior to the review. A total of 106 patients were identified. Imaging used to evaluate SCD was a 0.55 mm collimation temporal bone HRCT with reconstruction of images parallel to the plane of the SCC. Temporal bone HRCT images were reanalyzed in order to evaluate the SCD in a standardized fashion among all the patients. We defined dehiscence as the complete absence of a portion of the bone overlying the SSC. Cases of thinning of the bony roof did not meet our criteria for dehiscence and were excluded from the study. Doubt of investigators about whether or not the SSC was really dehiscent also resulted in exclusion from the study. Linear measurement of the dehiscence was performed by two separate investigators blinded to the patient’s clinical history and audiometric tests. The mean of the two values was then used.

In clinic, whenever SCD was suspected, we used a standardized form (Table 1) to evaluate the totality of SCDS signs and symptoms reported in this study. These patients were also systematically sent for an audiogram, a VEMP testing and a temporal bone HRCT. Each patient’s audiometric evaluation was assessed using pure-tone audiometry to obtain air conduction and bone conduction thresholds. Bone conduction was masked and measured in the supranormal range (-5 and -10 dB). ABGs were calculated for each frequency from 250 to 4,000 Hz. The VEMP was evoked using a Blackman Tone Burst Generator with a rarefaction tone burst at 500 Hz. All patients with radiologic confirmation of SCD had been explained the risks and benefits of the procedure and asked whether or not they wanted to undergo this surgical treatment in light of their symptoms.

**Table 1:** Clinical Signs and Symptoms of Operated and Non-Operated Patients at Superior Canal Dehiscence Diagnosis

	Number of patients		Ratio of patients (%)		P-value
	Operated group (N = 28)	Non-operated group (N = 34)	Operated group (N = 28)	Non-operated group (N = 34)	
Symptoms					
Cochlear symptoms					
Hypoacousis	17	22	60.7	66.7	0.091
Tympanophony	12	1	42.9	3	< 0.001
Autophony	28	9	100	27.3	0.004
Tinnitus	23	19	82.1	57.6	0.004
Pulsatile tinnitus	25	7	89.3	21.2	0.011
Phonophobia	26	8	92.9	24.2	0.008
Aural fullness	26	10	92.9	30.3	0.019
Other forms of hyperacousis					
Footstep sound	16	1	57.1	3.0	< 0.001
Eating sound	16	1	57.1	3.0	< 0.001
Oculophony	17	4	60.7	12.1	< 0.001
Sense of vibration	15	1	53.6	3	< 0.001
Vestibular symptoms					
Vertigo	9	7	32.1	2.2	0.012
Vertigo with effort	9	2	32.1	6.1	0.009
Imbalance/dizziness	25	16	89.3	48.5	0.024
Motion dizziness	17	9	60.7	27.2	0.017
Tullio phenomenon	16	3	57.1	9.1	0.002
Oscillopsia					
At rest	12	2	42.9	6.1	0.008
With walking	10	0	35.7	0	< 0.001
With effort	14	0	50.0	0	< 0.001
Signs					
Tuning fork at malleolus	10	2	35.7	6.1	0.003
Vertigo induced by pneumatic speculum	12	5	42.9	15.2	0.010
Valsalva manoeuvre	13	1	46.4	3	< 0.001
Hennebert	1	0	3.6	0	0.157

### Statistical analysis

Operated and non-operated group symptoms were compared using the Student’s *t*-test, while a Pearson’s Chi-squared test was used for the prevalence of each symptom. Audiograms were compared using a two-way repeated measures analysis of variance with one intrasubject factor (group) and one intersubject factor (frequency). All statistical analyses for this study were performed using SPSS 21. Statistical significance was defined as P < 0.05.

## Results

Between 2007 and 2012, a total of 106 patients had a temporal bone HRCT requested in order to confirm or rule out SCD. Among these, 62 were found to have SCD of which 28 underwent a surgical treatment, while 34 chose not to. All surgical procedures were performed by the senior author (IS) using the middle cranial fossa approach.

### Patient characteristics

There were 15 males and 13 females undergoing surgery with a mean age at diagnosis of 44 years (range: 27 - 60 years). Twenty-one patients presented unilateral SCD, which was present on the right side for 10 cases and on the left side for 11 cases. Seven patients had bilateral SCD, one with only the right and six with only the left ear operated. All patients had mixed symptomatology with both cochlear and vestibular symptoms. Signs and symptoms present at diagnosis are summarized in Table 1.

In our series of 62 patients presenting SCD on HRCT scan, 34 (17 males and 17 females) chose not to undergo surgery. Mean age in this group was 50 years (range: 22 - 74 years). Eleven patients had only cochlear symptoms while the 23 remaining presented a mix of cochlear and vestibular symptomatology (Table 1).

### Signs and symptoms

Operated patients showed a statistically significant difference in the number of cochlear symptoms, compared to non-operated patients (6.6 per patient (range: 5 - 11) vs. 2.4 (range: 0 - 5); P < 0.001). Moreover, when symptoms are analyzed individually, the operated group showed a statistically significantly higher proportion of patients having each cochlear symptom, except for hypoacousis (P = 0.091). All 28 patients (100%) who underwent surgery showed autophony at SCDS diagnosis, which makes it the most prevalent cochlear symptom in this group. However, presence of autophony was only found in 27.3% (nine of 34) of non-operated patients. Other forms of hyperacousis, such as hypersensitivity to one’s footsteps sounds (one of 34) or eating sounds (one of 34), tympanophony (one of 34) and sense of vibration (one of 34) were almost absent in the non-operated group.

Operated patients had a mean number of 3.4 vestibular symptoms out of the eight vestibular symptoms we assessed (range: 1 - 6). There was no statistically significant difference (P = 0.059) when compared to non-operated group whose average number of vestibular symptoms per patient was 1.1 (range: 0 - 4). When vestibular symptoms were analyzed individually, patients from the operated group were shown to experience them more frequently, except for vertigo (P = 0.068). None of the non-operated subjects (0 of 34) presented oscillopsia with effort or oscillopsia with walking, compared to 50.0% and 35.7% of patients in the operated group, respectively.

Four signs were investigated in all patients: hearing tuning fork at malleolus, vertigo induced by pneumatic speculum, vertigo induced by Valsalva manoeuvre, and Hennebert sign. The mean number of positive signs was 1.1 (range: 0 - 3) in operated group, compared to 0.2 in the non-operated group (range: 0 - 2). There was a statistically significant association between the presence of each sign and the need for a patient to undergo surgery (P < 0.001), except for the Hennebert sign (P = 0.157).

### Audiograms

**Figure 1:**
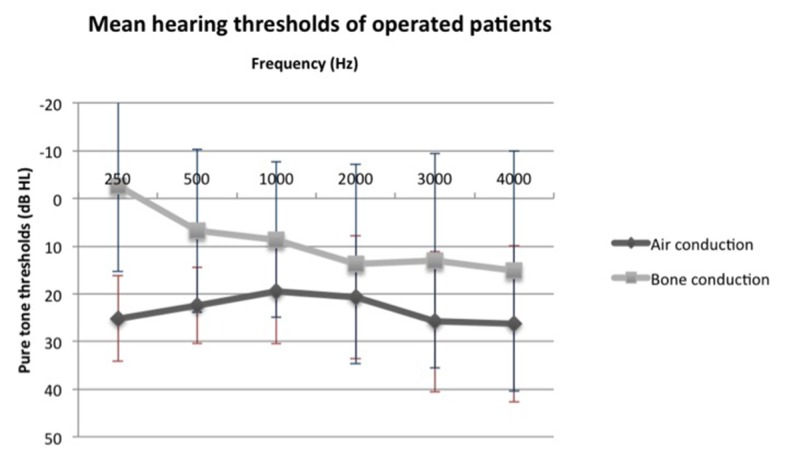
Mean air and bone conduction thresholds of operated patients before surgery.

**Figure 2:**
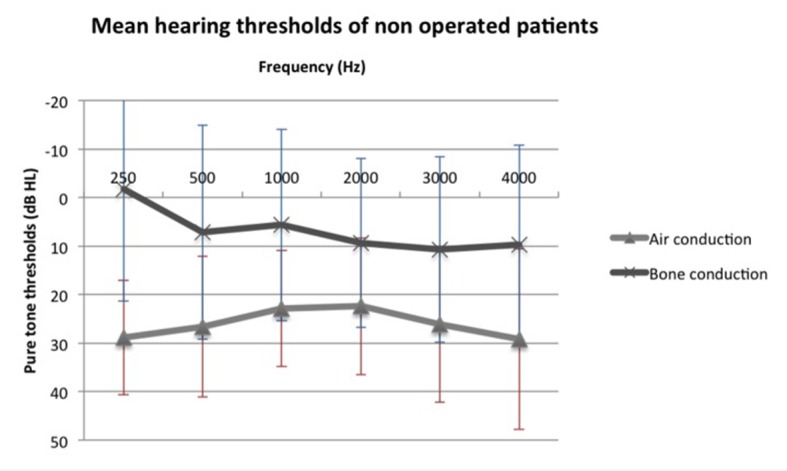
Mean air and bone conduction thresholds of non-operated patients.

We compared air and bone conduction thresholds between the two groups at 250, 500, 1,000, 2,000 and 3,000 Hz (Fig. 1, 2). Results showed no statistically significant difference regardless of the tested frequency (P > 0.05). Likewise, ABG at every frequency showed no significant difference between the two groups (P_250 Hz_ = 1.000, P_500 Hz_ = 0.387, P_1,000 Hz_ = 0.684, P_2,000 Hz_ = 1.000 and P_3,000 Hz_ = 1.000).

### VEMPs testing

Mean preoperative cVEMP threshold for the operated group was 62.4 dB (range: 50 - 75 dB, median: 65 dB), while the non-operated group showed a mean threshold of 70.6 dB (range: 50 - 90 dB, median: 65 dB). We found no statistically significant difference in the cVEMP thresholds elicited by the operated and non-operated group (P = 0.051).

### Dehiscence size

The size of the dehiscence was measured by two observers on reformatted HRCT scan images. It ranged from 2.0 to 8.0 mm with a mean size of 4.7 mm for operated patients. Standard deviation was 1.2 mm. Average dehiscence size of non-operated patients was 3.8 mm, ranging from 1.3 to 7.7 mm with a standard deviation of 1.8 mm. There was a statistically significant difference between the two groups (P = 0.421).

## Discussion

Manifestations of SCDS include a wide array of cochleovestibular signs and symptoms. Even though the exact physiological explanation behind this significant variability is still not completely understood, otolaryngologists are getting more familiar with this diagnosis. A sharper understanding of this clinical entity has led surgeons to develop newer surgical techniques in order to offer patients the lowest risks with the highest benefits. Middle cranial fossa (MCF) approach and plugging of the SSC has been the first and most studied technique. Postoperative improvement or resolution of symptoms such as sound- and pressure-induced vertigo, autophony, and hypoacousis has been consistently described in literature [1, 7, 14, 15]. Despite the positive surgical outcomes of SSC plugging, it is commonly accepted that most of the patients with SCD do not require treatment [1, 16, 17], even though an attempt to report this precise proportion has never been made. The conservative management rationale is likely based on one of the principal precepts of medical ethics, “Primum non nocere”. In fact, immediate complications encountered after MFC include epidural hematoma in some cases requiring surgical management [18], seizures [19] and CSF leaks [20]. The majority of patients report some degree of dizziness for several weeks to several months after SCD repair; however, in some patients, this vestibular hypo function can be persistent [21]. Also, early and delayed facial nerve palsy, although rare complications, can develop following craniotomy [22-24]. One of the proposed mechanisms includes thermal or mechanical injury to an underlying dehiscent geniculate ganglion. Interestingly, the prevalence of the latter has been reported to be higher in patients with SCD as opposed to without [16].

In our study, patients electing surgery not only showed significantly more cochlear symptoms, compared to the group choosing a conservative approach, but also variations in the nature of symptoms. Autophony, for instance, was present in all of the operated cases but only in 27.3% of the patients who preferred a conservative approach, potentially making it a marker of severity in SCD. Likewise, all forms of hyperacousis were underrepresented in the group refusing surgical treatment. Therefore, the fact that all operated patients suffered from at least one form of hyperacousis suggests its role as a determining factor impacting patients’ quality of life and decision to undergo surgery. Key vestibular symptoms were oscillopsia with effort and with walking which were exclusively found in the operated cohort affecting 50.0% and 35.7% of operated patients, respectively.

Several signs in favor of a superior canal dehiscence have been shown useful to screen for SCD [21, 25]. In this study, Valsalva and pneumatic speculum induced vertigo as well as hearing tuning fork at malleolus were present in a small proportion of non-operated patients compared to operated patients (3.0%, 15.2% and 6.1% vs. 46.4%, 42.9% and 37.4%, respectively). Regarding the audiogram results, ABGs, as well as air and bone conduction seem to be similar between the two groups. Likewise, cervical VEMP thresholds of both cohorts were comparable. Since its first description, a lot of research has been done to study the correlation between the size of the superior canal dehiscence and the various clinical features of the syndrome. Pfammter et al found that patients with larger dehiscence’s (≥ 2.5 mm) tend to present a mixed symptomatology while smaller dehiscence’s (< 2.5 mm) were associated to either cochlear or vestibular symptoms [5]. Chien et al on the other hand did not demonstrate a significant association between the dehiscence size and the number of signs and symptoms. A previous study conducted by our group showed that only the number of cochlear symptoms was influenced by the dehiscence size [12]. The relationship between the size of the dehiscence and the need for one to undergo surgery was never addressed before. Our results showed that patients with larger dehiscence’s more often than not needed a surgical treatment. Nevertheless, one could question the clinical significance of a 0.9 mm value difference between the two groups. As this is the first study to look at this relationship, more studies will be needed to confirm these results.

Providing patients with a complete explanation of the risks and benefits inherent to surgical canal repair is of utmost importance, as the ultimate decision regarding treatment choice will lie in their hands. A patient’s rationale for accepting or declining surgical treatment is an important unstudied variable in understanding the heterogeneity of use of this intervention. The reasons to decline surgery may be influenced by multiple factors (emotional, fear of surgery, time of recovery, etc.), but the decline in quality of life is most probably the driving factor. We can hypothesize that these patients may benefit from a different or simply less invasive surgical approach. In a recent review on the progression of surgical techniques used to repair SSC dehiscence, Shaia’s group summarized recent findings regarding transmastoid, endoscopic middle cranial fossa and transcanal approaches [13]. The latter and newest technique relies on the “third mobile window” to justify that reinforcement of any one of the three windows might improve SCDS. Transcanal round window obliteration has shown excellent success in the resolution of SCD induced symptoms even though long-term results remain to be studied. Given the minimally invasive nature of this procedure, its use may be beneficial in patients with mild quality of life impairment and when there is hesitation in choosing to undergo surgery or not.

### Strengths and limitations

A major strength of this study is the systematic assessment of signs and symptoms using a standardized form among all the patients with a suspected dehiscent superior canal. Surgery and conservative management has also been offered to all patients along with an exhaustive explanation of risks and benefits. One limitation however is the absence of a QOL assessment. In fact, although an objective evaluation is an important component in an SCDS questionnaire, every patient’s experience and symptomatology are often subjective. Prospective studies on a larger cohort of SCDS patients using established preoperative and postoperative QOL surveys would allow baseline QOL comparison among patients.

### Conclusion

A key concept is that despite the presence of SCD, not all patients will require surgical treatment. The purpose of this study was to raise common factors among patients declining surgery in order to better define this SCDS subpopulation. Our group was the first, to our knowledge, to compare a cohort of operated and non-operated patients presenting SCD. Numerous factors can be taken into account when assessing the need of a patient to undergo plugging or resurfacing surgery. Among the patients who decline surgery, some symptoms as footsteps and eating hyperacousis, tympanophony and oscillopsia with effort or walking are almost absent. On the other hand, presence of Valsalva/pneumatic speculum induced vertigo and hearing tuning fork at malleolus were all positively correlated with choosing surgery. Regarding the audiogram results, ABGs as well as air and bone conduction tend to be similar between the two groups. Likewise, cVEMP thresholds of both cohorts were comparable. We strongly believe that a good characterization of SCD subgroups of patients could eventually allow for a better understanding of the mechanisms through which a similar bony defect over the SCC displays interpersonal variability and help answer questions regarding SCD management. However, long-term, possibly multicenter, studies with larger populations will need to be conducted in order to do so.
